# Breakthrough in the high-pressure structures of Ba based on full exploitation of aperiodic symmetry

**DOI:** 10.1107/S2052252517001427

**Published:** 2017-02-23

**Authors:** Elena V. Boldyreva

**Affiliations:** a Institute of Solid State Chemistry, Russian Academy of Sciences, ul. Kutateladze 18, Novosibirsk 128, Russian Federation; b Novosibirsk State University, ul. Pirogova 2, Novosibirsk 90, Russian Federation

**Keywords:** high-pressure barium phases, incommensurately modulated structures, Ba IVb, atomic density waves, host–guest structures

## Abstract

A breakthrough in the high-pressure structures of Ba based on a full exploitation of aperiodic symmetry is discussed in the general context of the ‘complex structures of simple metals’.

Crystal structures of elements at high pressure (HP) are crucial for understanding the fundamental physics of condensed matter under extreme conditions and the basics of chemical bonding (Degtyareva, 2010 ▸; Steurer, 2014 ▸). Most elemental metals adopt simple face-centred cubic, body-centred cubic or hexagonal close-packed crystal structures under ambient conditions. Recent decades have seen the discovery that metals undergo phase transitions under elevated pressure, leading to an intriguing variety of extremely complex crystal structures. In many cases, these structures are unexpected within classical theoretical frameworks and they have been dubbed ‘complex structures of simple metals’. A considerable portion of these complex structures fall within the classification of host–guest type structures, typical for K, Na, Ba, Sr, As, Sb, Rb, and Bi. These structures are usually too complex to be solved from powder X-ray diffraction data and pose considerable difficulty for single-crystal diffraction data. The latter is largely due to the often imperfect nature of diffraction data from these structures. Despite numerous reports of their existence, no primary experimental data accompanied by precise systematic analysis have yet been presented in any publication, supplementary material or database.

Understanding complex phenomena requires high-precision data, resolving the finest details of the experimental picture. High-pressure crystallography has now reached a level where diffraction data allow the solution of HP structures to a similar accuracy as under ambient conditions. The resulting HP data are very robust and even sufficient to solve structures with *n*-dimensional (*n* > 3) periodicity. Finding true regularity in the *n*-dimensional arrangement of atoms can be done reliably using modern computing algorithms, avoiding groundless speculation and misleading interpretations of phenomena.

The paper by Arakcheeva *et al.* (2017 ▸) in ths issue of **IUCrJ** addresses the most complex case of an HP ‘complex metal structure’, the modification of the barium element structure Ba IVb, which has been solved for the first time at six different pressures from 16.5 to 19.6 GPa. Referring again to the required quality of data and their reliability, I note that the authors, in contrast to previous publications, have managed to perform all experiments under hydro­static compression conditions. This starts with the authors growing a single crystal at 15 GPa and subsequently collecting diffraction data from the other phases of the Ba metal. It evidently ensures the thermodynamic stability of all studied structures. Details of the analysis, parameters of the displacement modulations and experimental diffraction data with reliability indices are available within the supporting information for each pressure point.

The authors consider critically two possible models for the host–guest type of structure: a composite (COMP) model and an incommensurately modulated (IM) model. Normally, experimental diffraction data allow one to directly recognize a composite structure, which is characterized by periodicity of both the host and guest components. One can immediately recognize a fit to the COMP model from the full experimental diffraction data set, if two separate groups of reflections, which belong to the host and guest components, can be found. In contrast, aperiodicity originates from the irrational ratio of one or more of their unit cell parameters. If the two groups of reflections have an intersection, then both IM and COMP models must be considered and refined accurately, in order to select the correct model. Such an analysis is presented by Arakcheeva *et al.* (2017 ▸) for the first time. Surprisingly, it is the IM model, which has never before been considered for host–guest structures, that gives a better fit to the experimental data obtained for Ba across the 16.5–19.6 GPa pressure range. The importance of the selection of the correct model becomes clear on the basis of the new result: the model reveals an atomic density wave arising from the nearest Ba–Ba distance in the channels and its pressure-dependent evolution (Fig. 1 ▸).

The authors mention one more intriguing consequence of selecting the correct model for host–guest structures. If the COMP model is assumed, the temperature/pressure-dependent vanishing of reflections related to the guest component leads to the conclusion that there is one-dimensional melting of this chain component, *i.e.* the dis­appearance of any interaction between both host/guest and guest/guest atoms. If, however, the IM model is adopted, this phenomenon is instead interpreted as the disappearance of satellite reflections, *i.e.* the crystal structure becomes periodic with a new equilibrium in the interactions between host and guest atoms. Hence different models lead to completely opposite conclusions. This shows once again how critically important the reliability of crystallographic data and their careful evaluation are for obtaining correct insight into the physical and chemical properties of compounds and materials.

The article additionally presents the solution of two slightly different Ba IVb phases and gives some hints for the critical review of the solution of previously published structures of the same compound at higher and lower pressures. One could expect that the model developed by Arakcheeva *et al.* (2017 ▸) can also be applied for the description of complex HP phases of other elements. This could lead to considerable changes in our understanding of the physics of these systems.

## Figures and Tables

**Figure 1:**
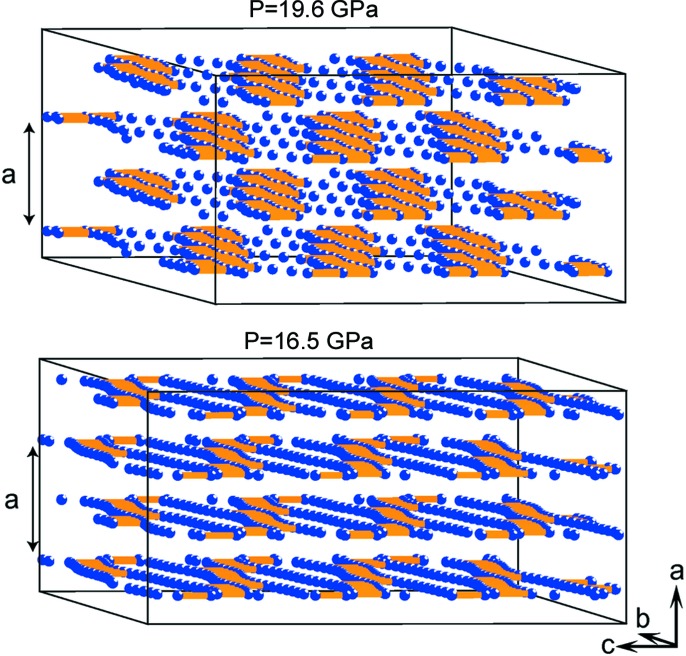
Illustration of the atomic density wave in the Ba IVb structure. The main structure modulation concerns the displacements of Ba atoms of the guest substructure along the *c* axis, *i.e.* along the channels formed by the host Ba atoms, which have been omitted to highlight the chessboard-like distribution of dense and sparse regions. The displacements induce variation in the Ba–Ba distances in the guest substructure. The orange sticks link atoms that sit within 2.90 and 3.8 Å of each other, showing a wave of dense and sparse arrangements of Ba atoms in the guest substructure.
